# An Interface Heterostructure of NiO and CeO_2_ for Using Electrolytes of Low-Temperature Solid Oxide Fuel Cells

**DOI:** 10.3390/nano11082004

**Published:** 2021-08-05

**Authors:** Junjiao Li, Jun Xie, Dongchen Li, Lei Yu, Chaowei Xu, Senlin Yan, Yuzheng Lu

**Affiliations:** 1 Department of Electronic Engineering, Nanjing Vocational Institute of Mechatronic Technology, Nanjing 211306, China; misslijunjiao@163.com; 2 School of Electronic Engineering, Nanjing Xiaozhuang University, Nanjing 211171, China; P2009035@njxzc.edu.cn (J.X.); 18090822@njxzc.edu.cn (C.X.); senlinyan@163.com (S.Y.); 3 Department of Electrical and Automation, Shandong Labor Vocational and Technical College, Jinan 250022, China; 2017033@njxzc.edu.cn; 4 Nanjing SolarU Energy Saving Technology Co., Ltd., Nanjing 210028, China; 230198941@seu.edu.cn

**Keywords:** nanomaterials, interface heterostructure, ionic conduction, band structure, built-in field, low-temperature solid oxide fuel cells

## Abstract

Interface engineering can be used to tune the properties of heterostructure materials at an atomic level, yielding exceptional final physical properties. In this work, we synthesized a heterostructure of a p-type semiconductor (NiO) and an n-type semiconductor (CeO_2_) for solid oxide fuel cell electrolytes. The CeO_2_-NiO heterostructure exhibited high ionic conductivity of 0.2 S cm^−1^ at 530 °C, which was further improved to 0.29 S cm^−1^ by the introduction of Na^+^ ions. When it was applied in the fuel cell, an excellent power density of 571 mW cm^−1^ was obtained, indicating that the CeO_2_-NiO heterostructure can provide favorable electrolyte functionality. The prepared CeO_2_-NiO heterostructures possessed both proton and oxygen ionic conductivities, with oxygen ionic conductivity dominating the fuel cell reaction. Further investigations in terms of electrical conductivity and electrode polarization, a proton and oxygen ionic co-conducting mechanism, and a mechanism for blocking electron transport showed that the reconstruction of the energy band at the interfaces was responsible for the enhanced ionic conductivity and cell power output. This work presents a new methodology and scientific understanding of semiconductor-based heterostructures for advanced ceramic fuel cells.

## 1. Introduction

Fuel cells efficiently convert the chemical energy of different fuels (e.g., H_2_, CH_4_) into electricity, avoiding the limitations of the Carnot cycle. Based on the electrolyte type, fuel cells can be classified in five groups: proton exchange membrane fuel cells, solid oxide fuel cells (SOFCs), molten carbonate fuel cells, alkaline fuel cells, and phosphoric acid fuel cells [1]. SOFCs are often used at high temperatures (700–1000 °C), making them the most promising candidates for clean energy since they do not require precious metal catalysts and their all-solid structure alleviates potential erosion [2]. High operating temperatures of SOFCs provide high ionic conductivity but also yield serious problems. The long-term stability of SOFCs is a great challenge. A traditional anode-supported SOFC may suffer from the apparent agglomeration of Ni particles in the Ni-YSZ anode (Ni-Y_2_O_3_ stabilized zirconia), and Sr can readily migrate from the La_0.6_Sr_0.4_Co_0.2_Fe_0.8_O_3-δ_ cathode to the electrolyte layer, yielding high interface resistance [3,4]. Additionally, it is difficult to monitor electrochemical behavior in a fuel cell at high temperatures [5,6]. To reduce the operating temperature of SOFCs, extensive research has focused on new materials. Zhu et al. reported the fabrication of a semiconductor-ionic fuel cell (SIFC) [7,8]. Composite materials made of perovskite semiconductors (e.g., SrFeO_3_ [9], Sr_2_Fe_1.5_Mo_0.5_O_x_ [10], La_0.6_Sr_0.4_Co_0.2_Fe_0.8_O_3-δ_ [11,12], and doped ceria [CeO_2_]) have been used to fabricate low-temperature electrolytes for SIFCs. Zhou et al. successfully applied pure SmNiO_3_ with electronic conductivity as an electrolyte for low-temperature SOFCs [13], which indicates the suitability of some semiconductor materials with electronic conductivity as electrolytes in fuel cells.

Ionic conductivity of cerium-based oxides can be improved by doping with rare earth materials, such as Gd [14] and Sm [15], or co-doping with two elements [16]. Although doped CeO_2_ has been considered to be an alternative electrolyte in SOFCs, electrolytes used in a fuel cell undergo chemical reduction due to H_2_ exposure, which results in deterioration of the open circuit voltage (OCV) and power output [17]. Recent results have suggested that specific surface strategies regarding CeO_2_ might be useful to overcome these challenges. Wang et al. introduced a logical design for non-stoichiometric CeO_2-δ_ based on undoped CeO_2_ [18]. They constructed a CeO_2−δ_@CeO_2_ core-shell heterostructure as an electrolyte for low-temperature SOFCs. A remarkable power output of 660 mW cm^−2^ was achieved at 550 °C. This was a simple and feasible new approach to low-temperature SOFCs with sufficient ionic conductivity. Xing et al. reported a proton shuttles in the CeO_2_/CeO_2−δ_ core-shell structure, exhibiting a unique proton transport mechanism in which the i-type CeO_2_ semiconductor is the core and the p-type CeO_2−δ_ semiconductor is the shell [19]. A maximum power density of 697 mW cm^−2^ was obtained based on the charged layers formed at the interface of the CeO_2−δ_/CeO_2_ heterostructure at 520 °C. This introduced a new generation of proton ceramic fuel cells. Liu et al. prepared a composite for insulating Sm_2_O_3_ and intrinsic p-type conductive NiO as an electrolyte of SOFCs [20]. The high total electrical conductivity of 0.38 S cm^−1^ and the corresponding power output of 718 mW cm^−2^ were achieved in the H_2_/air atmosphere at 550 °C. The results illustrate that interfacial ionic conduction between these two phases is a dominant factor that yields significant enhancement in proton conductivity. Very recently, Cai et al. developed bulk-heterostructure electrolytes based on Ce_0.8_Sm_0.2_O_2−δ_ and SrTiO_3_ to reduce the operational temperature of SOFCs [21]. They achieved a high peak power density of 892 mW cm^−2^ and an open circuit voltage of 1.1 V at 550 °C. They explained that a Schottky junction is formed in the cell, which can overcome the short-circuit issue.

Based on the above-mentioned strategy of interfacial ionic conductivity between two phases and the potentially attractive properties of CeO_2_, we discovered in this study that a semiconductor CeO_2_-NiO heterostructure has both proton and oxygen ionic conductivities. To improve the electrochemical properties of fuel cells, we added Na_2_CO_3_ into the composite to fabricate a new CeO_2_-Na-NiO electrolyte material. Further optimization of this study may be a useful approach to improve the electrochemical performance of SOFCs.

## 2. Materials and Methods

### 2.1. Synthesis of CeO_2_-NiO and CeO_2_-Na-NiO Powders

All chemicals and reagents used were of analytical grade and purchased from Aladdin. The solution was prepared by mixing 5 g of an NiO and CeO_2_ mixture (weight ratio of 3:1) in deionized water (200 mL), which was then stored at 80 °C for 10 h under constant stirring. Concentrated HNO_3_ was gradually added to the solution until the NiO was completely dissolved. The product was calcined at 700 °C in air for 2 h and then ground to obtain CeO_2_-NiO powder.

Additionally, a Na_2_CO_3_ solution with a stoichiometric ratio was added to the above-mentioned NiO and CeO_2_ solution. Following the same process explained above, the CeO_2_-Na-NiO sample was synthesized. BaZr_0.1_Ce_0.7_Y_0.2_O_3−δ_ (BZCY) was obtained by the method described in [22].

### 2.2. Characterization

The powder X-ray diffraction (XRD) pattern of the as-prepared sample was recorded with a Cu-Kα (λ = 1.54060 Å) source on a Bruker AXS D8 advanced X-ray diffractometer (Bruker Corporation, Billerica, Massachusetts, Germany). The microstructure and morphology of the as-prepared materials were analyzed by scanning electron microscopy (SEM; ZEISS Merlin SEM, Oberkochen, Germany) operating at 15 kV. Transmission electron microscopy (TEM) was performed on a Philips CM12/STEM device with an accelerating voltage of 120 kV. X-ray photoelectron spectroscopy (XPS) data were collected on a Physical Electronics Quantum 2000 device (Al Kα X-ray source) for surface and chemical analyses. The fuel cell performance and electrochemical properties were recorded using an electronic load instrument (IT8511, ITECH Electrical Co., Ltd., Shanghai, China) at 530 °C. The flow rate of H_2_ and air were 100 and 150 mL min^−1^, respectively, at a pressure of 1 atm. Electrochemical impedance spectroscopy (EIS) was employed to investigate the polarization characteristics of the electrode. The EIS measurements were tested under OCVs using an electrochemical workstation (Gamry Instruments, Reference 3000, Warminster, PA, USA) in a frequency range of 0.1–1.0 MHz. To further analyze the mechanism during the electrode process, we used ZSimpWin software (Version 3.1, Echem software, Leeds, UK) to fit the impedance spectra.

### 2.3. Cell Construction and Measurement

The LiNi_0.8_Co_0.15_Al_0.05_O_2−δ_ (LNCA; Tianjin Bamo Sci. & Tech. Joint Stock Ltd., Tianjin, China) electrode powder was mixed with terpineol to form a slurry, which was then brushed on one side of the Ni foam and dried in an oven at 120 °C for 1 h to obtain the Ni-LNCA electrode. The as-prepared CeO_2_-NiO and CeO_2_-Na-NiO composites were sandwiched between two Ni-LNCA pieces and pressed under a load of 250 MPa to fabricate a single-cell sample with an effective area of 0.64 cm^2^ and a thickness of approximately 2 mm. The as-fabricated single cells were symmetrical structures of Ni-LNCA/CeO_2_-NiO/LNCAL-Ni or Ni-LNCA/CeO_2_-Na-NiO/LNCAL-Ni. The flow rates were controlled at 100–120 mL min^−1^ for H_2_ and 150–200 mL min^−1^ for air at a pressure of 1 atm. All samples were tested at 530 °C after sintering at 600 °C under air for 0.5 h.

## 3. Results

### 3.1. Crystalline Structure and Morphology

**Figure 1:**
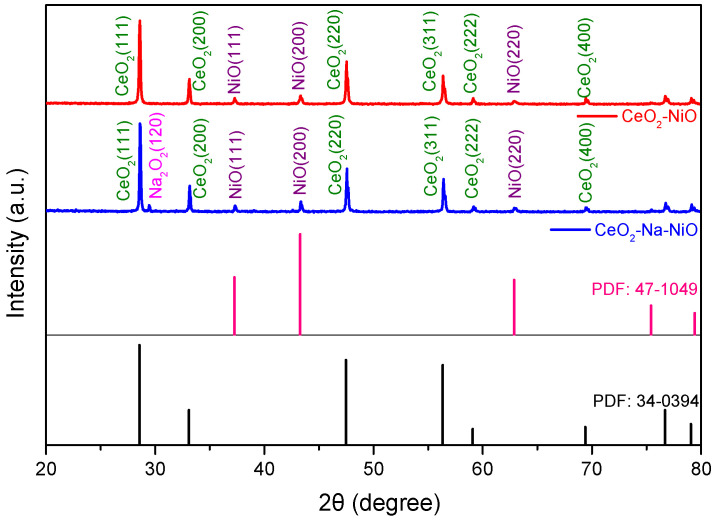
XRD patterns for the prepared CeO_2_-NiO and CeO_2_-Na-NiO.

Figure 1 shows XRD patterns of the as-prepared CeO_2_-NiO and CeO_2_-Na-NiO samples. The characteristic peaks were assigned to CeO_2_ (PDF#34-0394) and NiO (PDF#47-1049), indicating the coexistence of CeO_2_ and NiO in the as-prepared CeO_2_-NiO heterostructure composite. In the CeO_2_-Na-NiO sample, the phase at 29.26° can be well assigned to Na_2_O_2_ (PDF#16-0270), indicating that Na can be found in the CeO_2_-Na-NiO sample. Any other peaks originating from a chemical reaction between CeO_2_ and NiO did not appear in the XRD pattern.

**Figure 2:**
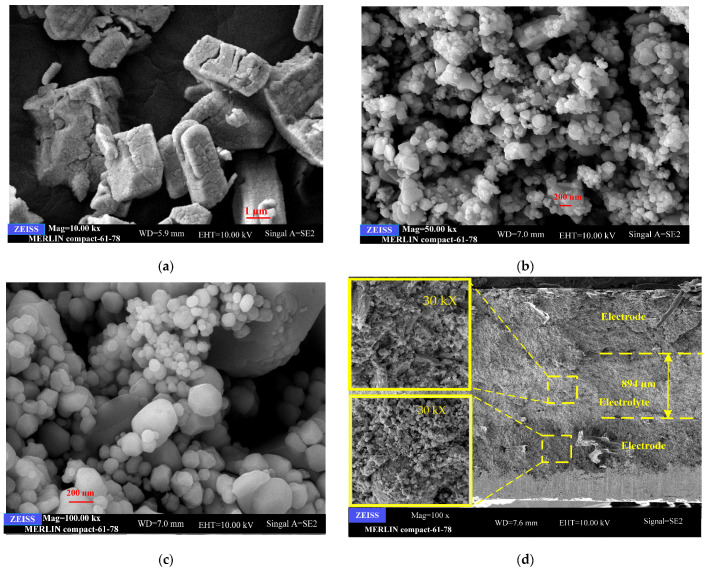
SEM images of (**a**) commercial CeO_2_; (**b**) CeO_2_-NiO; (**c**) CeO_2_-Na-NiO; (**d**) the cross-sectional SEM graph of CeO_2_-Na-NiO.

The morphology and microstructure of the commercial CeO_2_ and the as-prepared CeO_2_-NiO and CeO_2_-Na-NiO are shown in Figure 2a–c. The SEM micrographs of the commercial CeO_2_ and the prepared CeO_2_-NiO were obtained at a magnification of 50 kX (Figure 2a,b), and a magnification of 100 kX was used for the CeO_2_-Na-NiO sample (Figure 2c). This allowed us to observe nanometer-sized features. The commercial CeO_2_ had particles between 200 and 900 nm (Figure 2a). The as-prepared CeO_2_-NiO and CeO_2_-Na-NiO composites had smaller particles of 50 to 200 nm (Figure 2b,c). The small grain size in the nanometer range and enhanced interconnections in the CeO_2_-NiO or CeO_2_-Na-NiO composites may have contributed to their better electrochemical performance [23]. Additionally, the surfaces of the larger CeO_2_ particles were coated with smaller NiO particles, forming a significant interfacial area between CeO_2_-NiO (Figure 2b,c). Figure 2d shows the cross-sectional SEM graph of CeO_2_-Na-NiO, demonstrating that the three layers of the device had an electrolyte layer of about 894 μm thick. The SEM image of electrolyte layer at higher magnification of 10 Kx is inset in Figure 2d, showing a dense structure. It can be also found that SEM image of electrode layer at higher magnification of 10 Kx which is inset in Figure 2d, indicating that the electrode materials are homogeneous particles.

**Figure 3:**
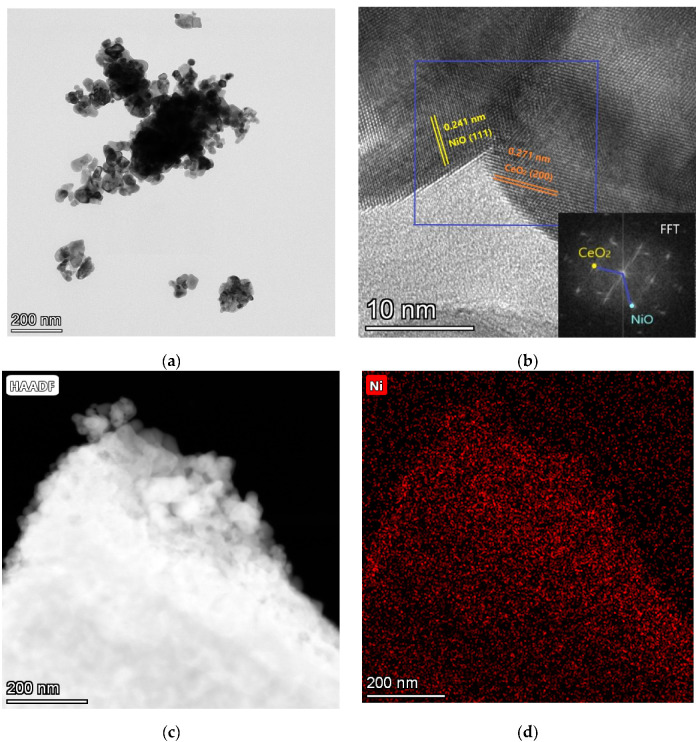

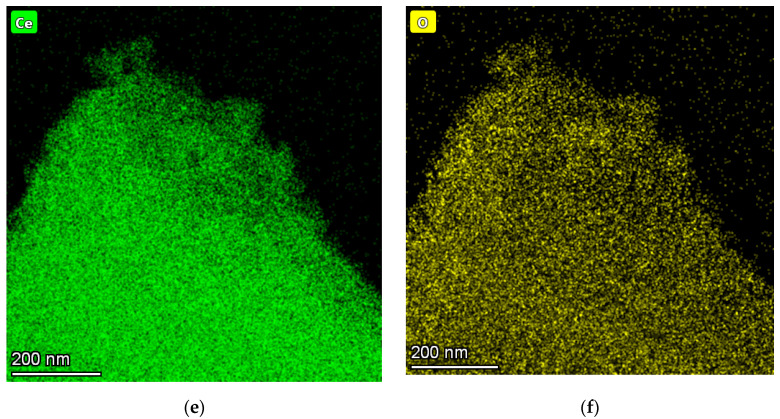
(**a**) TEM and (**b**) HRTEM images of CeO_2_-Na-NiO, with the inset in (**b**) giving the fast Fourier transform of corresponding HRTEM; (**c**) HAADF-TEM image and corresponding EDS maps of CeO_2_-Na-NiO for (**d**) Ni, (**e**) Ce, and (**f**) O.

The hetero-interfaces between the CeO_2_ and NiO were identified using high-resolution transmission electron microscopy (HR-TEM) (Figure 3). This may be an underpinning factor behind the enhanced ionic conductivity, since the hetero-interfaces between CeO_2_ and NiO may provide fast channels for both ion and proton conduction. Well-defined crystalline fringes with lattice spacing of 0.271 nm corresponding to the (200) crystal plane of CeO_2_ and 2.41 nm corresponding to the (111) crystal plane of NiO were observed, further shown by the fast Fourier transform (FFT) pattern (inset Figure 2b) which is in line with XRD results. Figure 3d provides the elemental mapping results from the HR-TEM test based on Figure 3c. The distribution of Ce, Ni, and O were clearly observed in the electrolyte materials, as showing in Figure 3e,f, elucidating that the Ce, Ni, and O elements are uniformly distributed over the region.

**Figure 4:**
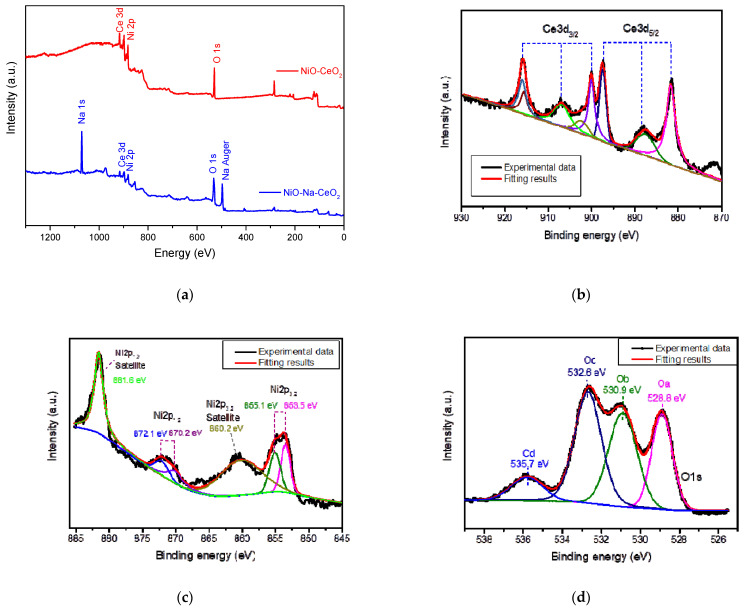
(**a**) XPS spectra of as-prepared CeO_2_-NiO and CeO_2_-Na-NiO, corresponding to survey scan, (**b**) Ce 3d spectrum, (**c**) Ni 2p spectrum, and (**d**) O 1s spectrum.

The surface chemical state of the as-prepared samples was analyzed using the XPS method (Figure 4). Ni, Ce, and O were detected in the CeO_2_-NiO sample, while Ni, Ce, Na, and O were present in the CeO_2_-Na-NiO sample (Figure 4a). The high-resolution XPS spectrum of Ce 3d is shown in Figure 4b. The vibration of Ce^4+^ peaked at a low binding energy and Ce^3+^ at higher binding energies. The Ce^4+^ peak at ~529.1 eV was assigned to the oxygen atoms in Ce(^+4^)-O, while the Ce^3+^ peak at 531.2 eV was assigned to oxygen-deficient regions at the interface (Figure 4d), which is related to their high ionic conductivities [19].

The Ni 2p XPS spectrum of the nanostructured NiO is shown in Figure 4c. The spectrum was divided into two edges due to spin-orbit splitting, namely 2p_1/2_ (~885–870 eV) and 2p_3/2_ (~869–845 eV) edges [24]. The main 2p line did not exhibit a significant blue shift compared to that of the corresponding single crystals. In addition, two main satellite structures, at ~1.5 and ~7.0 eV on the high-binding energy side of the main line, were present for both 2p_1/2_ and 2p_3/2_ edges, and their positions did not differ significantly from those of NiO single crystals [25]. The most important and striking difference between the XPS line shape of the nanostructured and single-crystal NiO was the observed main line bonding.

The peak of Na^+^ (1071.40 eV) was also found on the surface since Na^+^ ions can diffuse toward the surface of a composite material. The locally diffused Na^+^ can attract nearby electrons [26], which can reduce electron mobility on the surface, yielding an effective reduction in the internal short-circuit current of the composite electrolyte.

### 3.2. Electrochemical Performance

**Figure 5:**
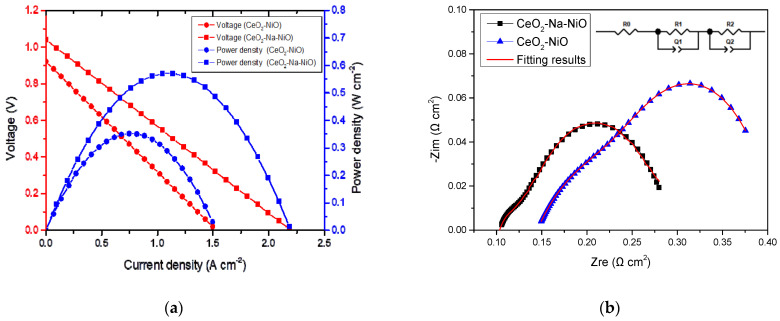
(**a**) Electrochemical performance of the fuel cells with CeO_2_-NiO and CeO_2_-Na-NiO at 530 °C; (**b**) EIS of Figure 2. NiO and CeO_2_-Na-NiO at 530 °C.

**Figure 6:**
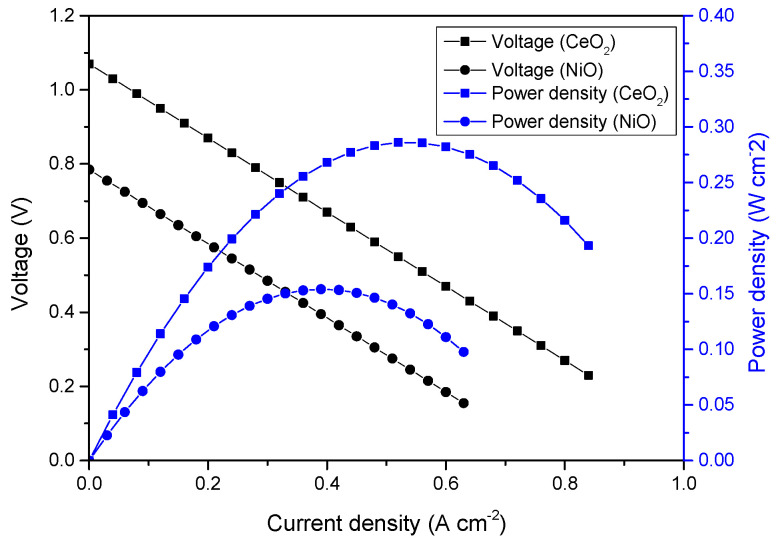
Electrochemical performance of the fuel cells with pure CeO_2_ and NiO at 530 °C.

The current-voltage (I−V) and current-power (I−P) characteristics of the fuel cells with CeO_2_-NiO and CeO_2_-Na-NiO interface heterostructures as electrolytes are shown in Figure 5a. A remarkable peak power density of 571 mW cm^−2^ was obtained for CeO_2_-Na-NiO, which is significantly higher than the 350 mW cm^−2^ obtained for CeO_2_-NiO at 530 °C. As a comparison, pure commercial CeO_2_ and NiO were also tested under the same conditions. NiO did not exhibit any considerable outputs for practical applications, and OCV values were below 1 V *(*Figure 6), indicating short-circuit issues. However, the composite of these two semiconductor materials showed enhanced power density. The output of the CeO_2_-NiO heterostructure fuel cell was 350 mW cm^−2^*,* and the corresponding OCV was 0.92 V, which illustrates minimal electronic short-circuit issues. Na^+^ in the CeO_2_-NiO composite improved both the power density (571 mW cm^−2^) and OVC (1.04 V), indicating that electronic transport in the electrolyte was suppressed. This experimental result was consistent with the XPS analysis.

This phenomenon differed to some extent from the state-of-the-art fuel cell technology. Great enhancements in power densities and OCV should originate from high proton and oxygen ionic conductivities in the electrolyte via the interface. This was demonstrated by the lower power densities obtained from the pure CeO_2_ and NiO and higher power densities obtained from the composite of CeO_2_-NiO and CeO_2_-Na-NiO. These as-analyzed interfaces enhanced ionic conductivities, as was also reported for other semiconductor materials [27,28,29].

### 3.3. Electrical Conductivity and Electrode Polarization

To investigate the conductivity mechanism of the fuel cell with the semiconductor composite electrolyte, we performed EIS measurements. Figure 5b shows the electrochemical impedance spectra of the CeO_2_-NiO and CeO_2_-Na-NiO samples under fuel cell operating conditions at 530 °C, with an equivalent circuit (R_0_(R_1_Q_1_)(R_2_Q_2_)) used to simulate the obtained results. In the equivalent circuit, R_0_ represents ohmic resistance of the electrolyte, R_1_ and R_2_ are polarization resistances, and Q is the constant phase element (C_PE_). The experimental results were simulated in ZSimpWin software, and the data are summarized in Table 1. The simulated results mainly show three contributions, i.e., one semi-circle with an additional small arc at high frequencies. The high-frequency side reflects the contribution of the grain resistance, the second intermediate frequency area is the contribution of the grain boundary resistance, and the third progress is the polarization resistance reflecting charge transfer behavior at low frequencies [30,31].

### 3.4. Mixed Oxygen-Ion-Proton Conducting Mechanism

**Figure 7:**
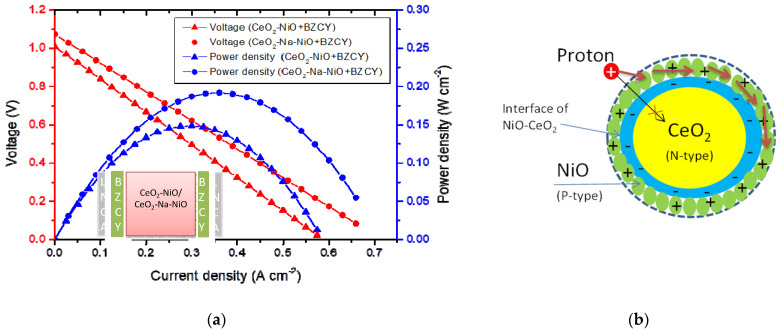

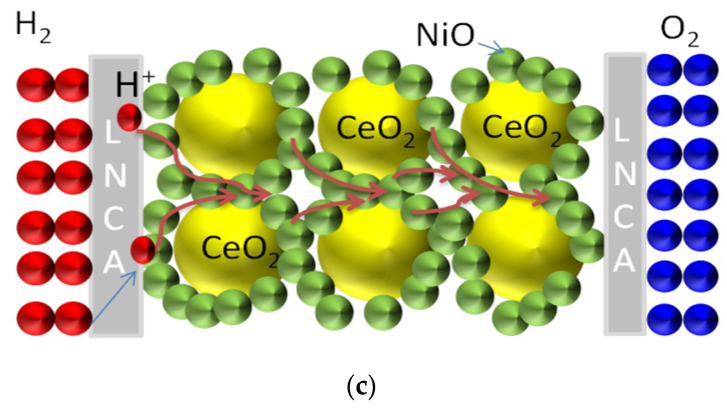
(**a**) Electrochemical performance of the fuel cells with only proton conductivity using BZCY at 530 °C; (**b**) the “proton shuttles” transport in the high-conducting region of the electrolyte membrane constituted by the interface heterostructure of CeO_2_-NiO; (**c**) charge separation at the interface of CeO_2_-NiO particle.

As discussed above, ionic conductivity plays a key role in cell performance, while electronic conductivity has an adverse effect. The significantly enhanced power output of the CeO_2_-NiO and CeO_2_-Na-NiO heterostructures could be attributed to a great enhancement in the ionic conductivity due to the interfacial effect since individual CeO_2_ or NiO samples did not exhibit good performance. The interface-enhanced ionic conductivity has also been found in other heterostructure composite materials [29,32]. We also found that proton conductivity can occur through CeO_2_-NiO and CeO_2_-Na-NiO electrolyte layers. The ions passed through the perfect bulk lattice, while the proton transport happened through the layer by the interface structure. This is different from the traditional bulk oxygen ion (O^2−^) conduction mechanism due to “proton shuttles”, which contribute to much better performance (Figure 7c). NiO is a p-type semiconductor [20], while CeO_2_ holds an n-type character [19]. Therefore, a p–n-type contact was constructed at the interface between CeO_2_ and NiO. A charge separation mechanism existed at the CeO_2_-NiO interface due to electron transfer from NiO to the CeO_2_ (Figure 7b). An electron depletion region formed at the NiO side of the interface and a corresponding electron accumulation region at the CeO_2_ side of the interface. Furthermore, the charge separation was additionally enhanced at the operating temperatures of the fuel cells. The positively charged layer in NiO prevented the proton from migrating to the depth of NiO and crossing the interface with CeO_2_ due to electrostatic repulsion (Figure 7b). Consequently, the proton transport was limited to the surface and a shallow layer near the NiO surface region. Due to a weaker H–O interaction and lower activation energy of proton diffusion in NiO, the proton transport was easier in NiO than in CeO_2_. Finally, due to the beneficial blocking effect of the positively charged layer in NiO, the “proton shuttles” performed the transport process in continuous high-conducting regions formed on the SOFC electrolyte membrane (Figure 7c). Martin and Duprez determined the oxygen and hydrogen surface diffusion on the oxide surfaces and pointed out that both oxygen and hydrogen can transport rapidly on the CeO_2_ surface [33,34].

Secondly, the NiO-CeO_2_ composites exhibited both proton and oxygen ionic conductivities. To prove the existence of proton conductivity in the CeO_2_-NiO and CeO_2_-Na-NiO composites, special cells were fabricated using BZCY in the configuration of Ni-LNCA/BZCY/x/BZCY/LNCA-Ni (x = CeO_2_-NiO, CeO_2_-Na-NiO), which can block the transport of O^2^^−^ and e^−^ (Figure 7a). Such special cells allowed only the proton transport through the electrolyte, contributing to the fuel cell output. The proton conductivity of the CeO_2_-NiO and CeO_2_-Na-NiO samples are shown as I–V and I–P characteristics in Figure 7a. The power densities of 148 mW cm^−2^ and 191 mW cm^−2^ were determined for CeO_2_-NiO and CeO_2_-Na-NiO with BZCY, respectively. The high current and power outputs confirm the considerable proton conductivity of the as-prepared CeO_2_-NiO and CeO_2_-Na-NiO samples. Bonano [35] and Maria [36] also provided other methods to block the transport of O^2−^ in the composites.

The proton conductivity (δ_iH_) was estimated from the slope of polarization curves in the ohmic polarization region as shown in Table 2.

δ_i_ = δ_Io_ + δ_IH_ (1)

where δ_i_ is the ionic conductivity, including both proton (δ_iH_) and oxygen ionic (δ_iO_) conductivities. The δ_i_ and δ_iH_ were estimated from the polarization curve (I−V) of the fuel cells as the linear part of the curve was known since the ohmic resistance was dominated by the electrolyte [37]. According to this method, the proton and oxygen conductivity were calculated as shown in Table 2. The δ_iH_ values of the CeO_2_-NiO and CeO_2_-Na-NiO with BZCY devices represented 37.3% and 29.7% of δ_i_, respectively. These results are in agreement with the outputs of the CeO_2_-NiO and CeO_2_-Na-NiO with BZCY devices. The outputs of 42.3% and 33.5% contributed to the proton conductivity because BZCY was used to block the O^−2^. The little discrepancy between these two datasets is acceptable considering the resistance of BZCY.

As discussed, ion conductivity includes both oxygen and proton contributions. Hence, the partial outputs for CeO_2_-NiO and CeO_2_-Na-NiO of 57.7% and 66.5% must have been caused by oxygen ionic conductivity. The results indicate that the ionic interfacial conduction may be a dominant ion conduction mechanism for the etched CeO_2_ electrolyte. The charge carriers of this interfacial conduction phenomenon were determined to contain oxygen ions and protons, as described above. The specific migration mechanism of oxygen ions and protons in CeO_2_-NiO or CeO_2_-Na-NiO electrolytes requires further investigation.

### 3.5. Mechanism of Blocking of Electron Transport

The question of how semiconductor interface heterostructures suppress electronic conductivity, which results in high ionic conductivity, needs to be clarified. As reported, the electronic conductivity has both positive and negative impacts on the performance of SOFCs with a semiconductor and ionic composite electrolyte [38]. The appropriate number of electrons in the heterostructure can enhance the triple phase boundary of both anode and cathode functional regions, which can greatly reduce polarization resistance [39]. In contrast, exorbitant electronic conductivity of the composite will induce a short-circuit issue, yielding low OCVs and power outputs. In this work, the semiconducting heterostructure was constructed for a novel electrolyte using a p-type (NiO) and n-type (CeO_2_) semiconductor. The device with the CeO_2_-Na-NiO heterostructure exhibited significantly better ionic conductivity and power output, accompanied by high OCVs at low temperatures. The working mechanism was based on a p–n heterojunction in the CeO_2_-NiO heterostructure membrane, which is a novel aspect of state-of-the-art SOFCs. The heterostructure was observed using the SEM and HR-TEM microscopy and gave insight into the interface conductivity of CeO_2_-NiO and CeO_2_-Na-NiO composites.

**Figure 8:**
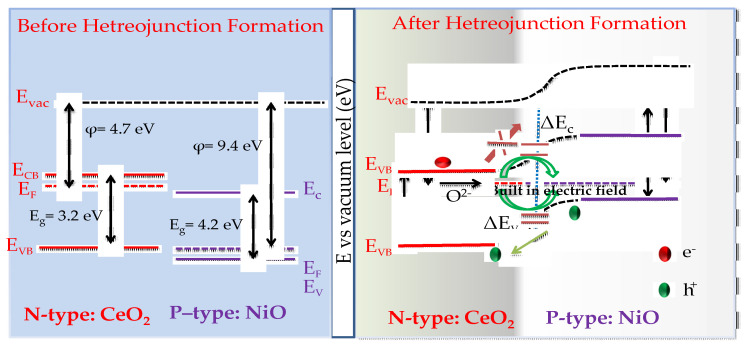
The band structure of CeO_2_-NiO heterostructure composites.

Generally, when two distinct particles or grains are interconnected, charge redistribution occurs as illustrated in Figure 8, where a desirable p–n heterojunction formed at the interface region between CeO_2_ and NiO due to different band offsets. This produced a local electric field and a potential gradient at the interfacial region [40]. Additionally, owing to different Fermi levels of CeO_2_ and NiO, the band inclined at the interface of the CeO_2_ and NiO heterostructure when two distinct particles or grains were interconnected. The charge transportation occurred from a higher to lower Fermi level to reach an equilibrium state at the interface. The redistribution of charges at the interface between CeO_2_ and NiO could have been due to the difference in Fermi level positions, valence bands, and bandgap energies. This resulted in the band incline in the CeO_2_ and NiO heterojunction. Different energy levels and similar Fermi energy levels of CeO_2_ and NiO induced an adjustment to the conduction band offset (ΔEc) and the valence band offset (ΔEv) at the interface to form potential barriers and a built-in electric field (Figure 8).

The principle is similar to that of solar photovoltaic cells. After the built-in electronic field is formed, it can block the electron transport through the electrolyte while charged species (e.g., O^2−^ or H^+^) can be easily moved from one side to the other. According to this new mechanism, it is easy to understand that the CeO_2_ and NiO heterostructure can indeed favor ionic transport for electrolyte function. As previously reported, the oxygen vacancies at the interface between CeO_2_ and NiO can be more stable and are easily produced with low formation of energy [41,42,43].

**Figure 9:**
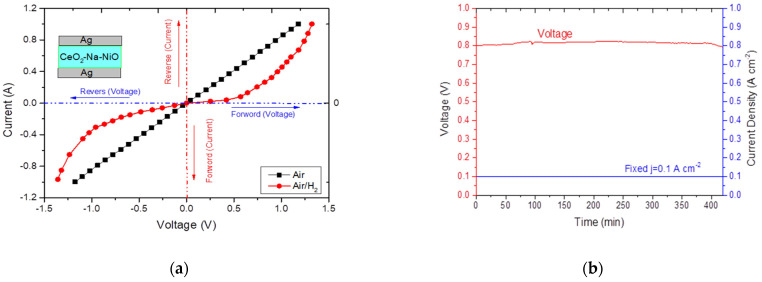
(**a**) the I−V characteristics under the bias measurements of the CeO_2_-Na-NiO heterostructure in air/air and in H_2_/air environments, and (**b**) the durability test result of the CeO_2_-Na-NiO sample at current density of 0.1 A cm^−2^ at 530 °C.

To prove the p–n heterojunction at the interface region between CeO_2_ and NiO, we prepared and tested a device with a configuration of Ag/CeO_2_-Na-NiO/Ag. The nonlinear rectification junction characteristic in the measured I–V curves reflected the existence of a built-in heterojunction [44,45], which blocked the electron transport through the device (Figure 9a). It is worth mentioning that the CeO_2_-Na-NiO sample was run stably for more than 7 h (Figure 9b), indicating that the CeO_2_-Na-NiO sample could function as an electrolyte for SOFCs with no obvious short-circuit problems during operation. Although stability was obtained in 7 h, long-term durability tests need to be further studied. Unfortunately, after operating for about 7 h, the voltage decreased rapidly, which indicates possible reduction of NiO to Ni. This phenomenon is consistent with the result reported by Liu et al. [20]. We will make further efforts to investigate the degradation mechanism and engineering technology to enhance the stability of the as-prepared device in the future.

## 4. Conclusions

In this study, a novel CeO_2_-NiO heterostructure for low-temperature SOFC electrolyte applications was successfully developed. The performance and conductivity of the device with the CeO_2_-NiO heterostructure were significantly enhanced compared to the individual NiO and CeO_2_. The introduction of Na^+^ ions into the composite electrolyte (CeO_2_-Na-NiO) reduced the mobility of electrons on the surface and further improved the overall performance. To establish the experimental descriptions, underlying mechanisms, and functionalities, we employed band alignment to explain the mechanism of ionic conductivity enhancement and the suppression of electronic conductivity. This was proven by the I–V characteristics under biased voltage, which resulted in a semiconductor behaving like a diode, indicating a junction effect in the CeO_2_-NiO fuel cell device. All these findings suggest that the semiconductor interface heterostructure charged reconstruction at the interface between n-type and p-type semiconductor materials as well as in the built-in electric field, playing a key role in the ionic conductivity enhancement and final excellent chemical performance. Therefore, the semiconductor interface heterostructure is a very promising approach for advanced low-temperature SOFCs.

## Figures and Tables

**Table 1:** EIS Fitted Data Using ZSimpWin Software @ 530 °C, Where R and C are in Ω cm^2^ and Yo[(S-s)^n^ cm^−2^], Respectively.

Sample	R_0_	R_1_	Q_1_	n	R_2_	Q_2_	n	Chi-Squared
CeO_2_-NiO	0.145	0.131	0.581	0.677	0.169	1.321	0.699	5.4 × 10^−4^
CeO_2_-Na-NiO	0.104	0.025	0.335	0.612	0.167	1.301	0.662	3.1 × 10^−4^

**Table 2:** The conductivities of the as-prepared materials at 530 °C.

Sample	δ_i_ (S cm^−1^)	δ_iO_ (S cm^−1^)	δ_iH_ (S cm^−1^)
CeO_2_-NiO	0.204	0.128	0.076
CeO_2_-Na-NiO	0.296	0.208	0.088

## Data Availability

The data present in this work are available on request from the corresponding author.
